# Understanding Dermatologists’ Acceptance of Digital Health Interventions: Cross-Sectional Survey and Cluster Analysis

**DOI:** 10.2196/59757

**Published:** 2025-05-21

**Authors:** Patrick Reinders, Matthias Augustin, Marina Otten

**Affiliations:** 1 Institute for Health Services Research in Dermatology and Nursing (IVDP), University Medical Center Hamburg-Eppendorf (UKE), Martinistraße 52, Hamburg, 20246, Germany, 49 40741024721

**Keywords:** dermatology, cross-sectional survey, cluster analysis, acceptability, eHealth, Germany, attitude, dermatologist, digital health intervention, teledermatology

## Abstract

**Background:**

Digital health interventions (DHIs) have the potential to enhance dermatological care by improving quality, patient empowerment, and efficiency. However, adoption remains limited, particularly in Germany.

**Objective:**

This study explores German dermatologists’ attitudes toward DHIs, clustering them by acceptance levels and analyzing differences in sociodemographics and current and future DHI use.

**Methods:**

We conducted a cross-sectional survey, randomly inviting 1000 dermatologists in Germany to participate. The questionnaire consisted of Likert scale items rating the acceptability of DHIs from 1 to 5. Items on the current and future use of DHIs were also included. Exploratory factor analysis was used to identify factors and reduce data as input for a 2-step clustering algorithm.

**Results:**

The survey with 170 dermatologists (mean age 50.8, SD 10.3 y; 74/167, 55.7% female) identified four factors through the exploratory factor analysis: (1) “Positive Expectancies and Acceptability of DHIs,” (2) “Dermatologists’ Digital Competencies,” (3) “Negative Expectancies and Barriers,” and (4) “Dermatologists’ Perspectives on Patients’ Acceptability and Competencies.” The analysis identified three distinct clusters: (1) Indecisives (n=69)—moderate intentions to use DHIs and moderate negative expectations toward them; (2) Adopters (n=60)—high intentions to use DHIs and high digital competencies; and (3) Rejectors (n=26)—low intentions to use DHIs and low digital competencies. Adopters were significantly younger, more often based in urban centers, and exhibited the highest adoption rates of DHIs compared to the other clusters. Across all clusters, inadequate reimbursement and perceived structural barriers were cited as significant challenges to DHI adoption. Still, only one-third of the Adopters used DHIs including teledermatology or artificial intelligence.

**Conclusions:**

Dermatologists in Germany exhibited varied levels of acceptance and readiness for DHIs, with demographic and structural factors influencing adoption. Addressing barriers such as reimbursement and investing in digital literacy could promote wider use, potentially reducing health inequalities by improving access to digital health care.

## Introduction

Dermatology is marked by the presence of prevalent chronic conditions, including acne, psoriasis, atopic dermatitis, and skin cancer [1,2]. In Germany, approximately 27% of adults have at least one dermatological condition requiring specialized care each year, resulting in long waiting times for patients [3,4]. Additionally, limited capacity also impacts comprehensive patient consultations, and the demographic shift could exacerbate these challenges [5-7].

Digital health interventions (DHIs) are proposed as one mitigating solution to sustain health care by enhancing the quality of care, efficiency, and empowering patients [8,9]. In this study, DHIs are defined as health services that use information and communication technologies for patients, consumers, or health care professionals [9,10]. In dermatology, numerous promising DHIs have already been developed [11], including artificial intelligence (AI) [12], teledermatology, diagnoses or monitoring tools [13-16], and patient-targeted apps for medication adherence [17]. The 2020 consensus guideline for teledermatology recommends its use for certain indications but also stresses the necessity for stronger evidence to evaluate benefits and risks [18]. Currently, no guidance exists for other apps.

Digitalization of health care in Germany lags behind that of other European nations [19]. Teledermatology, for example, is less frequently adopted in Germany [20]. The complexity of the situation is grounded in the intricacies of the German health care system, encompassing aspects such as missing interoperability, missing incentives for physicians, and high standards of data protection [21,22].

Apart from the system perspective, the reluctance of medical professionals to embrace digital health may be another potential barrier for the implementation [21,22], but can also function as a facilitator. Their acceptability can compensate for low patient demand or financial resource limitations and can additionally improve patient adherence to these interventions [23-26]. Dermatologists’ overall acceptability of DHIs appears to be lower than that of patients with a dermatological condition [27]. Nevertheless, it is noteworthy that dermatologists differ in their perspectives and adoption of DHIs, shaped by various factors including younger age, practicing in urban settings, positive prior experiences with DHIs, or the social influence of colleagues [28-30].

The objective of this study was to explore the acceptability and current and future use of DHIs among dermatologists. Additional objectives included identifying and describing clusters of dermatologists based on their acceptability and examining differences between these clusters and sociodemographic variables, as well as the current and future use of DHIs.

Decision makers or developers of DHIs can use these results to tailor strategies for a successful implementation.

## Methods

The consensus-based Checklist for Reporting of Survey Studies and the Checklist for Reporting Results of Internet E-Surveys were applied [31,32]. The full checklist is displayed in Checklist 1.

### Study Design and Questionnaire

An anonymous cross-sectional survey among German dermatologists was conducted in June 2022. The questionnaire was based on existing questionnaires from the literature, and in particular, on preconducted focus groups among dermatologists, patients, and nurses (not yet published). The focus groups dealt with the acceptability, barriers, and facilitators of DHIs in dermatology using the Unified Theory of Acceptance and Use of Technology [33,34]. The questionnaire development resulted in 27 items related to the acceptability of DHIs in general (eg, “I could imagine using more digital applications for the care of my patients”). Five items on the potential future use of specific DHIs (eg, “medication reminder application”) and four items on the benefits and costs of the nationwide eHealth Strategy (eg, “The eHealth Strategy has strengthened patient care”) were added to the survey. All items were answered on a 5-point Likert scale (5=strongly agree; 1=strongly disagree). The survey also included 10 items about dermatologists’ current use (>1 per wk) of available DHIs (eg, live-interactive teledermatology or electronic doctor letters) with a simple “check all that apply” option.

In addition, sociodemographic information covering age, sex, type of employment (eg, working in an outpatient clinic or medical practice), and postcode of the clinic or practice were collected. The postcode was used to assign additional regional data to participants (eg, urban or rural areas). The questionnaire was tested with 5 dermatologists of the Institute for Health Services Research in Dermatology and Nursing (German: Institut für Versorgungsforschung in der Dermatologie und bei Pflegeberufen). The final questionnaire is provided in Multimedia Appendix 1.

The offline version of this survey was designed to fit two DIN-A4 pages, while the web-based version was adapted to six screens or pages.

### Study Population, Recruitment, and Data Entry

In order to conduct an exploratory factor analysis (EFA; statistical analysis), we aimed for a minimum sample size of 150 dermatologists [35]. To reach the required sample size, the questionnaire was randomly distributed to 1000 members of the German Dermatological Academy (German: Deutsche Dermatologische Akademie [DDA] with ~3500 active dermatologists) in June 2022. The DDA comprises members of the Federal Association of German Dermatologists (German: Berufsverband der Deutschen Dermatologen), representing dermatologists from medical practices, and of the German Dermatological Society (German: Deutsche Dermatologische Gesellschaft), representing dermatologists from both outpatient clinics and medical practices. Dermatologists were asked to participate via email (Unipark), receiving one reminder or offline via mail on paper. The web-based survey was an open survey (no password protection). Additionally, participants could review their responses before submission. The survey was voluntary, and no incentives were offered. The survey was terminated after 8 weeks. The paper-based data were digitized, double-checked, corrected, and combined with the data of the web-based version. To prevent multiple entries by a single dermatologist in the web-based survey, participation was tracked using Unipark’s cookie system. To identify and eliminate further duplicate entries from the web-based and offline survey, demographic information and postcodes were cross-referenced and thoroughly checked. No duplicate entries were identified.

### Ethical Considerations

The study was submitted to the local ethics committee and was waived due to its noninvasive and anonymous nature. The study was reviewed and approved by the Lokale Psychologische Ethikkommission am Zentrum für Psychosoziale Medizin (LPEK), under the reference LPEK-0407. The study was conducted in accordance with Good Scientific Practice and the Declaration of Helsinki [36,37]. Due to the anonymous nature of the study, informed consent was not collected. Participants received no compensation for their participation and had the option to opt out of the survey at any time.

### Statistical Analysis

After conducting a descriptive analysis of all sociodemographic and geographic parameters, an EFA including all 27 items related to acceptability was performed for two reasons: (1) for data reduction by identifying underlying factors, also commonly used and recommended prior to running a cluster analysis; and (2) to use the underlying factors to closer describe and explore dermatologists’ acceptability toward DHIs [38,39].

Adequacy of the EFA for factor analysis was assumed with a Kaiser-Meyer-Olkin criterion (KMO) ≥0.8 and Bartlett’s test of sphericity <0.05. All factors with an eigenvalue ≥1.0 were considered. Criteria for retaining an item were a substantial factor loading (>|0.4|) on one factor and no strong cross-loading onto other factors. Cross-loading was defined as loadings (>|0.3|) on another factor and a difference of at least 0.2 between the loading on the main factor and the strongest loading on any other factor [40]. Internal consistencies of the factors were measured by Cronbach ɑ, where values >0.7 are acceptable, >0.8 are good, and >0.9 are considered excellent.

To identify distinct clusters of dermatologists that differ in their acceptability, a combination of hierarchical and K-means cluster analysis was applied. The mean scores (using Likert scale ratings: 1–5) of the factors were used as input variables for the 2 approaches. The hierarchical cluster approach was used to identify the optimal number of clusters. Subsequently, a K-means cluster analysis was conducted with this optimal number. To test on differences between the identified clusters based on mean scores, a one-way ANOVA was performed. In order to create a profile of the identified clusters, first, each item of the EFA (acceptability items) was binary coded (1=strongly agree or agree; 0=neither, disagree, strongly disagree). Then, a descriptive analysis was conducted, analyzing each item for each cluster. Next, chi-square tests were applied for categorical variables to identify differences in sociodemographic parameters, as well as current and potential future use of DHIs between clusters. In case more than 20% of cells had a count below five, Fisher’s exact tests were used. For continuous variables, one-way ANOVAs were used. A significance level of .05 was applied. Missing rates were reported for all variables. No imputation methods or weighting methods were applied.

## Results

### Participant Characteristics

In the survey, 170 dermatologists participated, resulting in a response rate of 17% (170/1000 invited dermatologists). In 131 (77.1%) cases, participation took place online. The online participation rate was 75.7%, calculated as the ratio of unique visitors who agreed to participate (131) to the total number of unique visitors who viewed the first survey page (173). Participants had a mean age of 50.8 (SD 10.3) years, 55.7% (74/167) were female, and for 84.2% (117/139) of participants, the practice or clinic was located in an urban county or city (Table 1). Missing data on all items were below 5% (9/170), except for regional variation (31/170, 18.2%) and outpatient clinic or medical practice (17/170, 10%).

**Table 1.** Sociodemographic characteristics of the total population and identified clusters.

	Total population^a^ (n=170)	Rejectors (Cluster 1; n=26)	Indecisives (Cluster 2; n=69)	Adopters (Cluster 3; n=60)	*P* value
Age group (years), n (%)					<.001
25‐39	24 (14.9)	0 (0)	5 (7.8)	17 (29.3)	
40‐49	33 (20.5)	2 (8.3)	14 (21.9)	13 (22.4)	
50‐59	71 (44.1)	9 (37.5)	39 (60.9)	18 (31)	
60 and older	33 (20.5)	13 (54.2)	6 (9.4)	10 (17.2)	
Missing	9 (5.3)	2 (7.7)	5 (7.2)	2 (3.3)	
Age, mean (SD)	50.8 (10.3)	58.8 (6.7)^b^^,c^	51.4 (7.9)^b^^,d^	46.9 (11.7)^b^^,^^d^	<.001
Sex, n (%)					.33
Female	74 (44.3)	12 (46.2)	25 (37.9)	32 (53.3)	
Male	93 (55.7)	14 (53.8)	41 (62.1)	28 (46.7)	
Missing	3 (1.8)	0 (0)	3 (4.3)	0 (0)	
Regional variation, n (%)					.01
Urban county or city	117 (84.2)	11 (61.1)	46 (82.1)	51 (91.1)	
Rural county	22 (15.8)	7 (38.9)	10 (17.9)	5 (8.9)	
Missing	31 (18.2)	8 (30.8)	13 (18.8)	4 (6.7)	
Outpatient clinic or medical practice, n (%)					<.001
Medical practice	108 (70.6)	26 (100)	54 (80.6)	28 (46.7)	
Outpatient clinic	45 (29.4)	0 (0)	13 (19.4)	32 (53.3)	
Missing	17 (10.0)	0 (0)	2 (2.9)	0 (0)	

a 15 participants could not be used in the cluster analysis, due to missing values of the used subscores.

b Different from Cluster 2 (Adopters); *P*<.05.

c Different from Cluster 1 (Indecisives); *P*<.05.

d Different from Cluster 3 (Rejectors); *P*<.05.

### Factors of Acceptability

The KMO criterion (0.92) and Bartlett’s test of sphericity (*χ*^2^_210_=2264; *P*<.001) revealed that the data were appropriate to conduct an EFA. Four factors with an eigenvalue >1 were identified (Table S1 in Multimedia Appendix 2). Cronbach ɑ values (0.93, 0.90, 0.73, and 0.80) indicated at least an acceptable internal consistency for all factors. The explained total variance of the EFA was 67.6%. A total of 6 items were excluded with a factor loading below 0.4. We named the factors as follows:

- Factor 1: “Positive Expectancies and Acceptability of DHIs” comprised of 10 items.
- Factor 2: “Dermatologists’ Digital Competencies” comprised of 5 items.
- Factor 3: “Negative Expectancies and Barriers of DHIs” comprised of 4 items.
- Factor 4: “Dermatologists’ Perspectives on Patients’ Acceptability and Competencies” comprised of 2 items.

The four mean scores of the factors ranged between 2.9 (SD 0.8) for Factor 4 and 3.5 (SD 0.8) for Factor 2 (Table 2). Ranging from 1 to 5, higher scores indicate a higher agreement with the items of a factor, except for Factor 3, where it is the other way around.

**Table 2.** Differences in identified mean scores on acceptability between the clusters.

	Total population (n=170), mean (SD)^a^	Rejectors (Cluster 1; n=26), mean (SD)	Indecisives (Cluster 2; n=69), mean (SD)	Adopters (Cluster3; n=60), mean (SD)	*P* value(ANOVA)
Factor 1: Positive expectancies and acceptability of DHIs^b^	3.4 (0.9)	2.0 (0.6)^c^^,d^	3.2 (0.6)^c^^,e^	4.1 (0.3)^c^^,e^	<.001
Factor 2: Dermatologists’ digital competencies	3.5 (0.8)	1.9 (0.7)^c,d^	3.4 (0.6)^c^^,^^e^	4.3 (0.4)^c^^,e^	<.001
Factor 3: Negative expectancies and barriers	3.4 (0.5)	3.8 (0.3)^c^^,d^	3.5 (0.4)^c^^,e^	3.0 (0.5)^c^^,e^	<.001
Factor 4: Dermatologists’ perspectives on patients’ acceptability and competencies	2.9 (0.8)	2.2 (0.7)^c^^,d^	2.7 (0.6)^c^^,e^	3.5 (0.6)^c^^,e^	<.001

a 15 participants could not be used in the cluster analysis, due to missing values of the used subscores.

b DHI: digital health intervention.

c Different from Cluster 2 (Adopters); *P*<.05.

d Different from Cluster 1 (Indecisives); *P*<.05.

e Different from Cluster 3 (Rejectors); *P*<.05.

### Clusters of Dermatologists According to Acceptability Levels

After exploring 2-, 3-, and 4-cluster solutions, the hierarchical cluster analysis recommended the 3-cluster solution as it provides the best fit based on the K-means evaluation. The performed ANOVA and subsequent post hoc tests revealed that all differences between the four mean scores of the three clusters were significant.

Cluster 1 (n=26, 16.7%) exhibited the lowest willingness (Factor 2: mean 1.9, SD 0.7) and digital competencies (Factor 2: mean 1.9, SD 0.7). Correspondingly, this cluster demonstrated the highest negative expectancies of DHIs (Factor 3: mean 3.8, SD 0.3) and low expectations regarding patients’ adequate competencies and willingness to use DHIs (Factor 4: mean 2.2, SD 0.7).

Cluster 2 (n=69, 44.5%) showed moderate positive expectancies toward DHIs (Factor 1: mean 3.2, SD 0.6), along with moderate digital competencies (Factor 2: mean 3.4, SD 0.6) and also moderate negative expectancies (Factor 3: mean 3.5, SD 0.4). Cluster 2’s expectations of patients’ intention to use DHIs and of patients’ digital competencies were low (Factor 4: mean 2.7, SD 0.6).

Cluster 3 (n=60, 38.7%) had higher intentions (Factor 1: mean 4.1, SD 0.3), greater competencies (Factor 2: mean 4.3, SD 0.4), and lower, though still moderate, negative expectancies (Factor 3: mean 3.0, SD 0.5) compared to Clusters 1 and 2. The cluster’s opinion about patients’ intention and competencies was higher, though still moderate (Factor 4: mean 3.5, SD 0.6).

Based on these differences, we will henceforth refer to Cluster 1 as Rejectors, Cluster 2 as Indecisives, and Cluster 3 as Adopters in the subsequent sections.

### Differences in Sociodemographic Information Between Clusters

The Adopters (mean age 46.9, SD 11.7 y) were significantly younger compared to both the Indecisives (mean age 51.4, SD 7.9 y; *P*=.03) and Rejectors (mean age 58.8, SD 6.7 y; *P*<.001). No significant differences in sex were observed among the clusters (*P*=.33). Notably, an association between regional variation (urban or rural) and the three clusters was identified (*P*=.01): the Rejectors had the highest proportion of participants from rural counties (7/18, 38.9%), while the Adopters had the highest percentage of participants from urban counties or cities (51/56, 91.1%). In addition, Indecisives (54/67, 80.6%) and Rejectors (26/26, 100%) performed their services primarily in medical practices, whereas Adopters (32/60, 53.3%) performed theirs in outpatient clinics (*P*<.001).

### Acceptability Profiles of the Clusters

**Figure 1.**
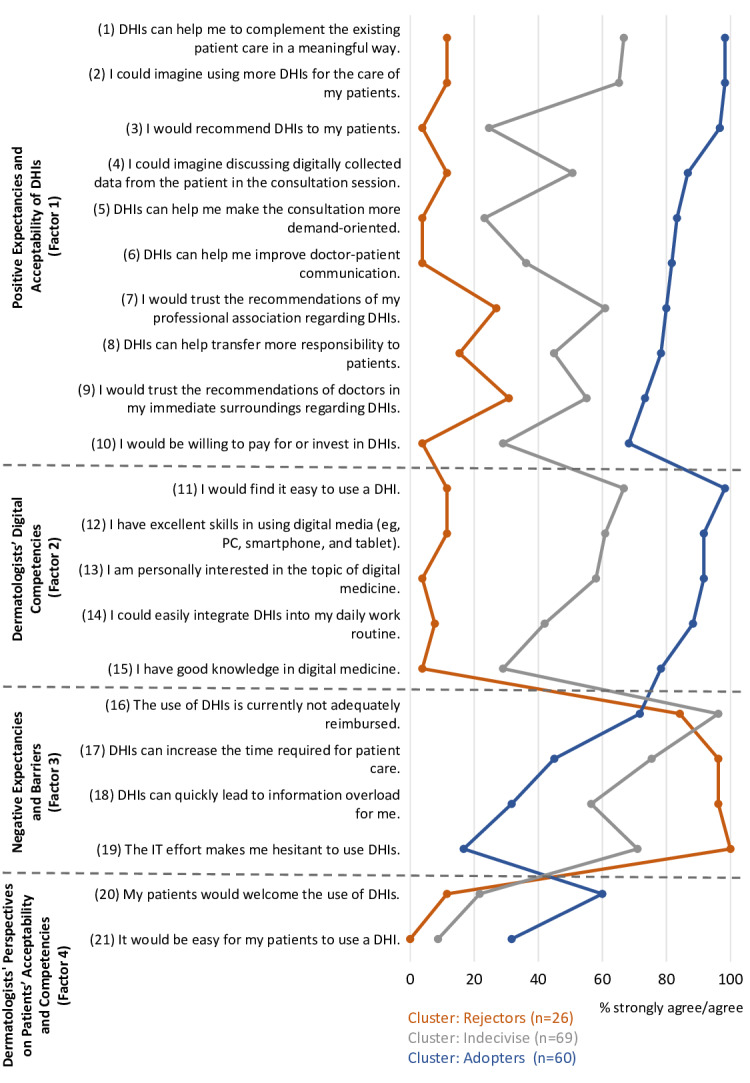
Cluster profile analyses on an item-by-item level. DHI: digital health intervention.

The analysis of individual items revealed distinct profiles of the three clusters on their attitude toward DHIs. The following paragraphs describe selected items for each factor and cluster. A comprehensive overview of all item ratings is presented in Figure 1.

Nearly all Adopters expressed openness to using (Item 2: 59/60, 98.3%) and recommending DHIs (Item 3: 58/60, 96.7%) in the future (Figure 1; Factor 1). However, a lower proportion was willing to pay for DHIs (Item 10: 41/60, 68.3%). They rated themselves highly in digital media skills (Item 12: 55/60, 91.7%) and expressed confidence in the easy integration of DHIs into daily routines (Item 14: 53/60, 88.3%; Factor 2). About 45% (27/60; Item 17) feared an increase in the time required for patient care due to DHIs, but only a minority saw the effort for IT as a burden (Item 19: 10/60, 16.7%; Factor 3). A majority agreed that patients would accept DHIs (Item 20: 36/60, 60.1%), while a minority agreed that patients would find using DHIs easy (Item 21: 19/60, 31.7%; Factor 4).

About two-thirds of Indecisives were willing to use DHIs in the future (Item 2: 45/69, 65.2%), but a minority would recommend (Item 3: 17/69, 24.6%) or invest in DHIs (Item 10: 20/69, 29%; Factor 1). Although the majority would find it easy to use a DHI (Item 11: 46/69, 66.7%), few considered themselves knowledgeable in digital medicine (Item 15: 20/69, 29%; Factor 2). The majority perceived IT effort as a burden (Item 19: 49/69, 71%), expressed concerns about DHIs increasing consultation time (Item 17: 52/69, 75.4%), and causing information overload (Item 18: 39/69, 56.5%; Factor 3). Only 21.7% (15/69) believed patients would welcome DHIs (Item 20), and 8.7% (6/69; Item 21) thought patients would find them easy to use (Factor 4).

Rejectors scored low in Factor 1, with the highest agreement rates in trust toward recommendations of other physicians (Item 9: 8/26, 30.8%) and professional associations (Item 7: 7/26, 26.9%; Figure 1; Factor 1). Only 11.5% (3/26; Item 12) agreed to have high competencies in digital medicine (Factor 2). Almost all Rejectors worried about increased time requirements for patients (Item 17: 25/26, 96.2%) and information overload (Item 18: 25/26, 96.2%). They perceived high IT effort as a barrier to DHIs (Item 19: 26/26, 100%; Factor 3). Only 11.5% (3/26; Item 20) believed patients would welcome DHIs, and none (Item 21: 0%) thought patients would find them easy to use (Factor 4).

Overall, ratings on items in Factors 1, 2, and 3 were distinctly different among the three clusters, with the exception that the majority within all clusters agreed that DHIs are inadequately reimbursed (Figure 1; Factor 3). For Factor 4, Indecisives and Rejectors showed similar low agreement rates on a per-item level (Figure 1).

### Assessment of the Nationwide eHealth Strategy

**Figure 2.**
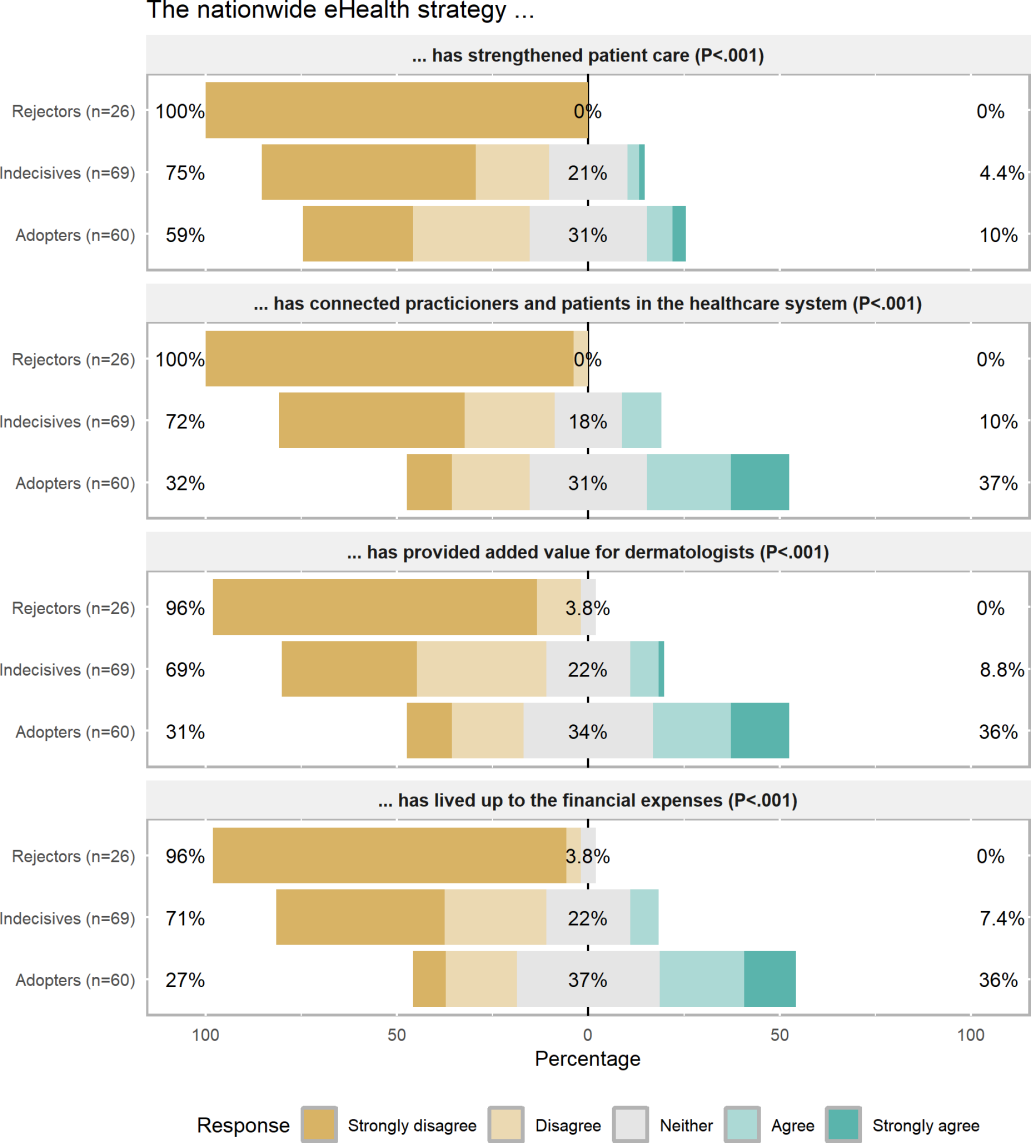
Assessment of the nationwide eHealth strategy by cluster*. P* values indicate the outcome of a chi-square test or Fisher exact tests.

In the assessment of the nationwide eHealth Strategy, most dermatologists across all clusters did not perceive the strategy as successful (Figure 2). A majority within each cluster concluded that the eHealth Strategy did not enhance patient care (Indecisives: 52/69, 75%; Adopters: 35/60, 58.3%; and Rejectors: 26/26, 100%).

However, significant variations could still be observed among the three clusters. All Rejectors disagreed on the strategy being effective in fostering connections between patients and providers (26/26, 100%). The Indecisives shared similar doubts (50/69, 72.4%). This behaved similarly with regard to the strategy being cost-effective (49/69, 71% disagreement for Indecisives; 26/26, 100% for Rejectors) and providing added value to dermatologists (48/69, 69.1% disagreement for Indecisives; 25/26, 96.2% for Rejectors). In contrast, the “Adopters” were more divided in their assessment: one-third of the cluster agreed with the statements, another third disagreed, and the remaining third was inconclusive. For example, 37.1% (22/59) agreed, 32.3% (19/59) disagreed, and 30.6% (18/59) were inconclusive on the statement regarding whether the strategy has connected patients and physicians.

### Current Use of DHIs Within Identified Clusters

**Figure 3.**
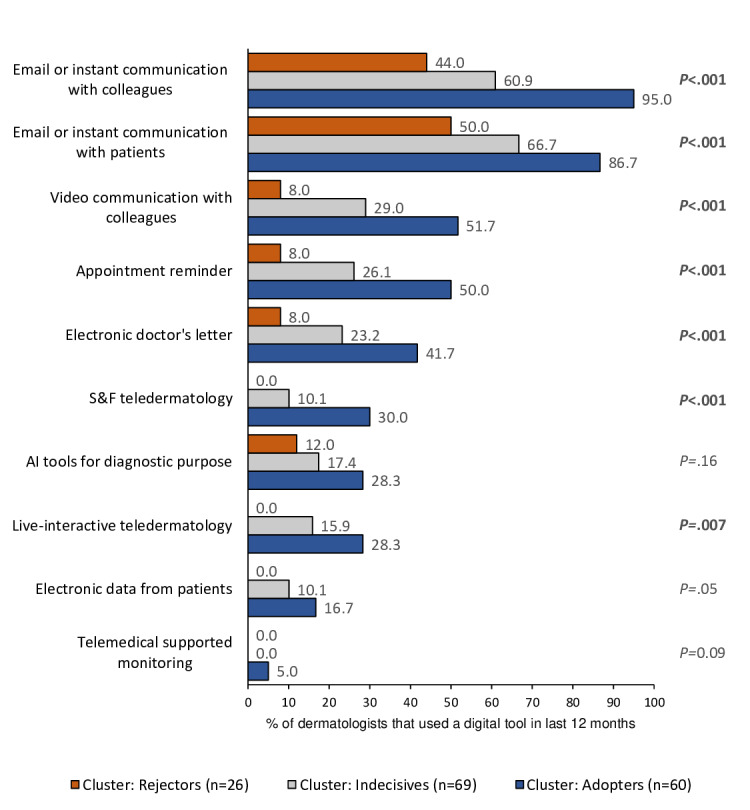
Use of digital technology in the last 12 months by clusters*. P* values indicate the outcome of a chi-square test or Fischer exact tests. AI: artificial intelligence; S&F: store-and-forward.

The use of common DHIs varied significantly between the three clusters, except for AI tools for diagnostic purposes (range: Rejectors 12%-Adopters 28.3%; *P*=.15), electronic patient data (range: Rejectors 0%-Adopters 16.7%; *P*=.05), and telemedical supported monitoring (range: Rejectors 0%-Adopters 5%; *P*=.09; Figure 3). The Adopters demonstrated the highest and the Rejectors the lowest utilization rates across all presented DHIs. A total of 95% (57/60) of the Adopters reported to communicate via email or instant messaging with colleagues, and 86.7% (52/60) used email to communicate with patients. In contrast, only 44% (11/25) and 50% (13/26) of the Rejectors engaged in these communication methods. Apart from email communication, less than half of the Adopters and one-third of the Indecisives used other DHIs. For example, “Appointment Reminders” were used by 50% (30/60) of the Adopters and 26.1% (18/69) of the Indecisives. The Rejectors did not use many of the DHIs at all, such as store-and-forward (0%) and live-interactive teledermatology (0%). Additionally, their utilization of AI for diagnostic purposes was very low (3/25, 12%).

### Future Intention to Use DHIs Within Identified Clusters

**Figure 4.**
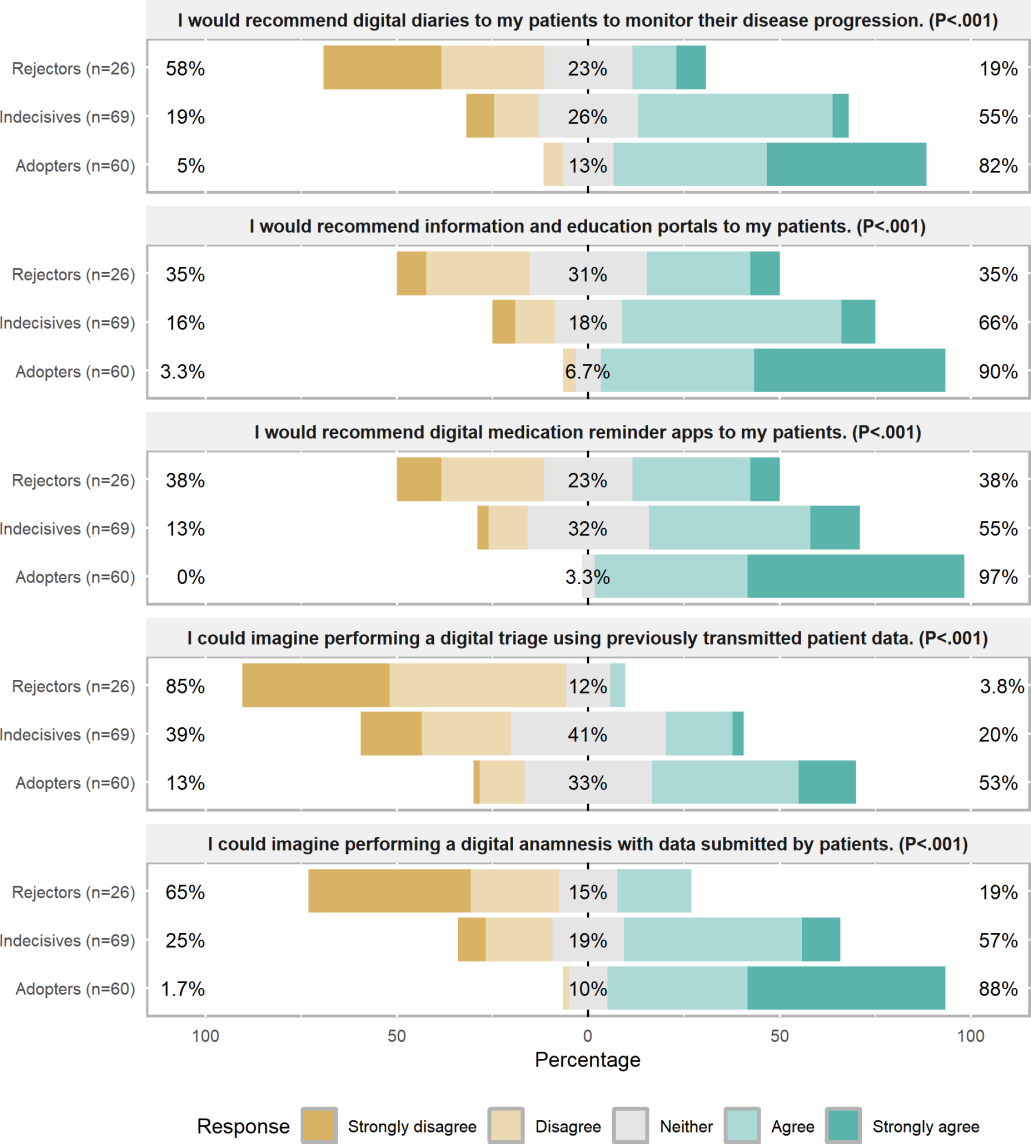
Potential future use of digital health interventions by clusters. *P* values indicate the outcome of a chi-square test.

When considering the potential use of DHIs for their patients in the future, significant differences were identified among the three clusters (Figure 4). Between 80% (48/60; patient diaries) and 97% (58/60; medication reminder) of Adopters were willing to use 4 out of 5 described interventions. In contrast, only 55% (38/69: medication reminders) to 66% (45/68; education portals) of Indecisives can imagine using or recommend specific DHIs more often than currently. Rejectors had low potential use rates ranging from 19% (5/26; for digital anamnesis) to 35% (9/26; for education portals). While a slight majority of Adopters (32/60, 53%) could envision using digital triage, 33% (20/60) were undecided. However, 20% (14/69) of the Indecisives could imagine using digital triage, while 41% (28/69) were undecided. In contrast, 85% (22/26) of the Rejectors disagreed on using digital triage in the future.

## Discussion

### Principal Findings

This study aimed to explore dermatologists’ acceptability of DHIs, as well as their current and potential future use. Further, clusters were identified according to their level of acceptability, and differences were examined between the clusters and sociodemographics, as well as DHI current and future use. The current use barely exceeded 30% for interventions like teledermatology, AI for diagnostic purposes, and electronic patient data, even among willing dermatologists.

Overall, acceptability was moderate but varied significantly among the identified clusters. We recognized a cluster of dermatologists with high acceptability (Adopters), another similar-sized cluster with moderate acceptability (Indecisives), and a third minority with low acceptability (Rejectors). Indecisives and Rejectors perceived negative expectancies following the introduction of DHIs (eg, workload increase) and mostly questioned patients’ willingness and competence to use them. Adopters were younger, and more commonly practicing in urban settings and outpatient clinics compared to Rejectors. Satisfaction with the nationwide eHealth strategy was generally low, although perspectives among Adopters were more divided. Current and future use of DHIs depended on the type of DHI and cluster, with Adopters showing higher rates.

Significant inequalities in DHI adoption between clusters indicate the presence of a second-order digital divide (disparities in digital competencies and acceptability toward DHIs) [41,42]. The more common adoption of DHIs among younger and urban dermatologists has already been identified [30]. This difference can contribute to existing health inequities by impacting access to health care and potentially even health outcomes [43]. It is crucial to closely monitor the impact of further DHI adoption in dermatology and address any potential health disparities.

In general, inadequate knowledge, as perceived in the survey, is a recognized barrier to the adoption of DHIs [22,44]. Integrating education on digital medicine into medical curricula and continuing medical education programs is, therefore, a potential approach to increase use and mitigate the digital divide [45]. As the health care system undergoes digital transformation, investing in digital health education is crucial [46].

The survey identified structural barriers to the widespread adoption of DHIs, including inadequate reimbursement and incentives, workload concerns, and IT infrastructure challenges. In addition, the inadequate nationwide eHealth infrastructure [47], as also noted by dermatologists in our survey, hinders the seamless integration of DHIs, for example, in teledermatology due to problems with electronic prescriptions and lack of access to patient records [21,48]. These factors may help explain why the adoption rate of innovative technologies, including teledermatology, remained below 30% even among willing adopters. To address these challenges, developers and decision makers must prioritize aligning the eHealth infrastructure with practitioners’ needs, as seen in other European countries [19]. Incentivizing physicians, alongside involving them in the development process more frequently to reduce concerns, can be a focus to increase DHI uptake [21].

The limited availability of DHIs may also contribute to the relatively low adoption rates, even among those categorized as Adopters. In Germany, for instance, store-and-forward teledermatology platforms primarily focus on diagnosis [15,16,49], with limited emphasis on triage or referrals [14] and follow-up of patients with chronic conditions [13]. This limitation restricts the broader use of the technology for different patient groups. Additionally, patient electronic health records remain incompletely implemented [19], and there is a shortage of patient apps capable of either generating (eg, heart rate) or supporting patients in generating (eg, patient-reported outcomes) health data. Conversely, the swift uptake of AI apps, even among those categorized as Rejectors, underscores the benefits of this technology for dermatologists [50]. Our research suggests that most Adopters and the majority of Indecisives express a desire to use and recommend specific DHIs more frequently, highlighting the need for increased availability of evidence-based DHIs.

The perspective that patients would not welcome or find DHIs easy to use aligns with evidence that over 70% of the German population lacks adequate digital health competencies [51]. Patients also demonstrate low to moderate acceptability toward DHIs [34,52-54]. Other research contradicts those findings, suggesting that patients with dermatological conditions even have a higher acceptability toward DHIs in comparison to dermatologists [27]. Nevertheless, physicians and nurses can provide vital guidance, given their significant influence on patients [23,25,26]. Notably, Adopters recognized this role, expressing a willingness to recommend DHIs despite acknowledging patients’ overall lack of digital competencies. This proactive stance should be complemented by health insurers, who also have a responsibility to promote DHIs and enhance digital literacy to ensure their appropriate utilization [55]. Such efforts are aligned with insurers’ interests in supporting cost-effective health care services [55, 56].

One key distinction we found is that Adopters are more commonly affiliated with a hospital-connected outpatient clinic, while the majority of the other clusters involve self-employed dermatologists in medical practices. This may explain the differences in opinions on costs, reimbursements, or investments. Physicians in outpatient clinics may be less concerned with IT costs and IT investments than physicians in private practices, as they do not have to organize or pay themselves [57].

Similar to other studies on acceptability toward digitalization, it is important to consider the Intention-Behavior gap when interpreting the survey results. Here, intending to adopt DHIs in the future may not guarantee increased actual adoption in the future [58]. Additionally, while an association has been observed between a group exhibiting high acceptability (Adopters) and their current adoption of DHIs, the cross-sectional nature of the study means that the direction of this association remains ambiguous and cannot be extrapolated into the future.

### Limitations

The survey has further limitations. Only 170 dermatologists participated out of over 6000 practicing dermatologists, of which 3500 were organized in the DDA and 1000 were invited [59]. Additionally, while dermatologists were randomly invited, our survey exhibited an overrepresentation of dermatologists in the medium age group (50–59 years), with younger dermatologists (40–49 years) and older dermatologists (60+) being underrepresented [59]. This observation is particularly significant considering the age differences identified between the clusters, where younger participants are more commonly affiliated with the Adopters cluster and older participants with the Rejectors cluster. The survey is also prone to voluntary participation, which might have attracted dermatologists with more extreme views on health care digitalization. Hence, the sizes of the clusters and other findings may not be fully generalizable to the real world. We also did not account for the potential integration of certain DHIs, such as the use of AI to preanalyze images submitted through store-and-forward teledermatology. Future research should therefore explore the impact of AI on perceptions and attitudes toward DHIs [54].

A factor analysis was conducted, as recommended, to reduce data dimensionality before performing the cluster analysis [39]. EFA was chosen instead of confirmatory factor analysis, as no theoretical assumptions about item interactions were made. The approach limits the comparability with other research on acceptability [33,34]. In addition, it is often recommended to conduct a confirmatory factor analysis following an EFA [60]. However, our sample size was not large enough to split the dataset to allow for a subsequent analysis. Despite this limitation, EFA calculations were deemed appropriate based on the KMO criterion and Bartlett’s test of sphericity, resulting in factors with acceptable internal consistency [40]. To validate the findings of our research, the results could be further explored by conducting qualitative interviews with dermatologists and using a mixed methods approach [61].

### Conclusions

The survey results indicate that dermatologists’ overall acceptability toward DHIs is moderate. However, a sizable cluster expresses a high willingness to incorporate these interventions and recommend them to patients. Two other distinct clusters exist: one indecisive about health care digitalization and another rejecting DHIs.

The observed variation in actual adoption rates among clusters suggests a nuanced relationship between willingness and the implementation of DHIs. Dermatologists who express willingness are more inclined to adopt DHIs more frequently. However, despite this willingness, overall use rates remain moderate. This may be attributed, in part, to several structural barriers, including inadequate reimbursement, insufficient IT infrastructure, limitations of the nationwide eHealth infrastructure, and the limited availability of DHIs.

A notable perception among dermatologists, especially those with low acceptability, is the perceived low patient willingness and competency regarding digital health. This points to efforts needed to enhance digital literacy among patients. It is imperative for all stakeholders in the health care system, including health insurers, to invest in initiatives to improve patient education and empowerment in digital health.

Additionally, prioritizing education for health care providers on digital health and involving them early in the development of DHIs could mitigate potential disparities in digital health.

Addressing all the mentioned barriers could improve the acceptability and use of DHIs among dermatologists, particularly among those who are indecisive, thereby enhancing the integration of DHIs to support patient care.

## Supplementary material

10.2196/59757 Multimedia Appendix 1 Overview of the items of the survey.

10.2196/59757 Multimedia Appendix 2 Factor loadings of items on the acceptability: results from the exploratory factor analysis.

10.2196/59757 Checklist 1 Checklist for Reporting of Survey Studies (CROSS)

## Abbreviations

AI: artificial intelligence
DHI: digital health intervention
EFA: exploratory factor analysis
KMO: Kaiser-Meyer-Olkin
