# Supplementary material for: Understanding Dermatologists’ Acceptance of Digital Health Interventions: Cross-Sectional Survey and Cluster Analysis
Source: JMIR Hum Factors. 2025 May 21;12:e59757. doi: 10.2196/59757 (PMC12118942; doi:10.2196/59757)
Supplement: Multimedia Appendix 1 [file humanfactors-v12-e59757-s001.docx]

## **Acceptability of digital health interventions**

**Explanation (German original):** Digitale Anwendungen meinen im Folgenden jegliche digitalen Technologien, die zur Erreichung von gesundheitsbezogenen Zielen eingesetzt werden können, z. B.: Telemedizin (Videosprechstunde, Telekonsile), elektronische Patientenakten, digitale Gesundheitsanwendungen (DIGA), Apps oder Web-Anwendungen für Ärzte und Patienten.

**Explanation (English translation):** In the following, digital applications refer to any digital technologies that can be used to achieve health-related goals, e.g: Telemedicine (video consultations, teleconsultations), electronic patient records, digital health applications (DIGA^[[1]](#footnote-1)^), apps or web applications for doctors and patients.

Each statement (Table S1) was rated on a 5-Point Likert scale:

**German original:** (5) Stimme völlig zu; (4) Stimme eher zu; (3) Unentschieden; (2) Stimme eher nicht zu; (1) Stimme gar nicht zu

**English translation:** (5) Strongly agree; (4) Agree; (3) Neither; (2) Disagree; (1) Strongly Disagree

**Table S1** Developed statements related to the acceptability of digital health interventions

| **Nr** | **English translation** | **German original** |
| --- | --- | --- |
| Quest. | To what extent do you agree with the following statements? | Inwieweit stimmen Sie den folgenden Aussagen zu? |
| 1 | I could imagine using more DHIs for the care of my patients. | Ich könnte mir vorstellen vermehrt digitale Anwendungen für die Versorgung zu nutzen. |
| 2 | I would be willing to pay for or invest in DHIs. | Ich wäre bereit für digitale Anwendungen zu zahlen bzw. in diese zu investieren. |
| 3 | I would find it easy to use a DHI. | Mir würde es leichtfallen eine digitale Anwendung zu nutzen. |
| 4 | I find it difficult to distinguish serious from dubious DHIs. | Ich finde es schwierig seriöse von unseriösen digitalen Anwendungen zu unterscheiden. |
| 5 | I could easily integrate DHIs into my daily work routine. | Ich könnte digitale Anwendungen einfach in meinen Arbeitsalltag integrieren. |
| 6 | My practice/clinic has the necessary infrastructure for the use of DHIs. | Meine Praxis/Klinik besitzt die nötige Infrastruktur für den Einsatz von digitalen Anwendungen. |
| 7 | Data collected from the patient should be easily transferable to my records. | Vom Patienten erhobene Daten müssten einfach in meine Unterlagen übertragen werden können. |
| 8 | I am concerned about misuse of collected data. | Ich mache mir Sorgen, dass erhobene Daten missbraucht werden können. |
| 9 | The IT effort makes me hesitant to use DHIs. | Der Aufwand für die IT schreckt mich ab digitale Anwendungen einzusetzen. |
| 10 | The use of DHIs is currently not adequately reimbursed. | Der Einsatz von digitalen Anwendungen wird derzeit nicht angemessen vergütet. |
| 11 | I would trust the recommendations of my professional association regarding DHIs. | Ich würde den Empfehlungen meines Berufsverbandes in Bezug auf digitale Anwendungen vertrauen. |
| 12 | I would trust the recommendations of doctors in my immediate surroundings regarding DHIs. | Ich würde den Empfehlungen von Ärzten aus meinem Umfeld in Bezug auf digitale Anwendungen vertrauen. |
| 13 | My non-physician colleagues in my practice/clinic would welcome the use of DHIs. | Meine nicht-ärztlichen Kollegen in meiner Praxis/Klinik würden den Einsatz digitaler Anwendungen begrüßen. |
| 14 | DHIs can quickly lead to information overload for me. | Digitale Anwendungen können für mich schnell zu einer Informationsüberflut führen. |
| 15 | DHIs can increase the time required for patient care. | Digitale Anwendungen können den zeitlichen Aufwand bei der Versorgung von Patienten erhöhen. |
| 16 | DHIs can help me make the consultation more demand-oriented. | Digitale Anwendungen können mir helfen die Sprechstunde bedarfsorientierter zu gestalten. |
| 17 | DHIs can help me improve doctor-patient communication. | Digitale Anwendungen können mir helfen die Arzt-Patienten-Kommunikation zu verbessern. |
| 18 | DHIs can help me to complement the existing patient care in a meaningful way. | Digitale Anwendungen können mir helfen die bestehende Patientenversorgung sinnvoll zu ergänzen. |
| 19 | I would recommend DHIs to my patients. | Ich würde meinen Patienten digitale Anwendungen empfehlen. |
| 20 | My patients would welcome the use of DHIs. | Meine Patienten würden den Einsatz digitaler Anwendungen begrüßen. |
| 21 | It would be easy for my patients to use a DHI. | Meinen Patienten würde es leichtfallen eine digitale Anwendung zu nutzen. |
| 22 | DHIs can make patients feel insecure about their condition. | Digitale Anwendungen können Patienten im Hinblick auf Ihre Erkrankung verunsichern. |
| 23 | DHIs can help transfer more responsibility to patients. | Digitale Anwendungen können helfen Patienten mehr Verantwortung zu übertragen. |
| 24 | I have excellent skills in using digital media (e.g., PC. smartphone and tablet). | Ich habe exzellente Fähigkeiten im Umgang mit digitalen Medien (z. B. PC, Smartphone, Tablet). |
| 25 | I am personally interested in the topic of digital medicine. | Ich interessiere mich persönlich für die Thematik der digitalen Medizin. |
| 26 | I have good knowledge in digital medicine. | Ich habe gute Kenntnisse in digitaler Medizin. |
| 27 | I could imagine discussing digitally collected data from the patient in the consultation session. | Ich könnte mir vorstellen vom Patienten digital erhobene Daten in der Sprechstunde zu besprechen. |

## **Assessment of the nationwide eHealth Strategy**

Each statement (Table S2) was rated on a 5-Point Likert scale:

**German original:** (5) Stimme völlig zu; (4) Stimme eher zu; (3) Unentschieden; (2) Stimme eher nicht zu; (1) Stimme gar nicht zu

**English translation:** (5) Strongly agree; (4) Agree; (3) Neither; (2) Disagree; (1) Strongly Disagree

**Table S2** Statements on the assessment of the nationwide eHealth Strategy

| **Nr** | **English translation** | **German original** |
| --- | --- | --- |
| Intro | The nationwide eHealth strategy (incl. telematics infrastructure and electronic patient records) up to and including today ... | Die bundesweite eHealth-Strategie (inkl. Telematikinfrastruktur und elektronische Patientenakte) bis einschließlich heute … |
| 1 | … has connected practitioners and patients in the healthcare system. | … hat für eine Vernetzung von Leistungserbringern und Patienten  im Gesundheitswesen gesorgt. |
| 2 | … has lived up to the financial expenses. | …ist den finanziellen Aufwendungen gerecht geworden. |
| 3 | … has provided added value for dermatologists. | …hat einen Mehrwert für Dermatologen erbracht. |
| 4 | … has strengthened patient care. | … hat die Patientenversorgung gestärkt. |

## **Current use of available DHIs**

**Table S3** Current use of available DHIs

| **NR** | **English translation** | **German original** |
| --- | --- | --- |
| Quest. | Which technologies do you use regularly (>1 per week) in your practice/clinic? (Check all that apply) | Welche Technologien nutzen Sie regelmäßig (>1-mal die Woche) in Ihrer Praxis/Klinik? (Zutreffendes ankreuzen) |
| 1 | Live-interactive teledermatology | Online-Videosprechstunde |
| 2 | S&F teledermatology | Zeitversetzte telemedizinische Verfahren (z. B. Derma2Go, AppDoc, OnlineDoctor) |
| 3 | E-Mail or instant communication with patients | Zeitversetzter Informationsaustausch mit Patienten (z. B. E-Mail, SMS) |
| 4 | E-Mail or instant communication with colleagues | Zeitversetzter Informationsaustausch mit Kollegen (z. B. E-Mail, SMS) |
| 5 | Video communication with colleagues | Kommunikation in Echtzeit zum fachlichen Austausch (z. B. Video-Konferenz) |
| 6 | Telemedical supported monitoring | Telemedizinisches Patienten-Monitoring |
| 7 | Electronic doctor's letter | Elektronischer Arztbrief |
| 8 | Appointment reminder | Elektronische Erinnerung an Termine |
| 9 | Electronic or online data from patients (e.g., …) | Elektronische oder Online-Daten von Ihren Patienten (z. B.) Apps, Wearables, Körperwerte) |
| 10 | AI tools for diagnostic purpose | Künstliche Intelligenz (KI)-Systeme zur Diagnostik |

## **Potential Future Use of DHIs**

Each statement (Table S4) was rated on a 5-Point Likert scale:

**German original:** (5) Stimme völlig zu; (4) Stimme eher zu; (3) Unentschieden; (2) Stimme eher nicht zu; (1) Stimme gar nicht zu

**English translation:** (5) Strongly agree; (4) Agree; (3) Neither; (2) Disagree; (1) Strongly Disagree

**Table S4** Potential Future Use of DHIs

| **Nr** | **English translation** | **German original** |
| --- | --- | --- |
| Quest. | To what extent do you agree with the following statements? | Inwieweit stimmen Sie den folgenden Aussagen zu? |
| 1 | I could imagine performing a digital anamnesis with data submitted by patients. | Ich könnte mir vorstellen in Zukunft eine digitale Anamnese mit zuvor übermittelten Patientendaten zu nutzen. |
| 2 | I could imagine performing a digital triage using previously transmitted patient data. | Ich könnte mir vorstellen in Zukunft eine digitale Triage mithilfe zuvor übermittelter Patientendaten durchzuführen. |
| 3 | I would recommend digital diaries to my patients to monitor their disease progression. | Ich würde meinen Patienten digitale Krankheitstagebücher zur Verlaufskontrolle empfehlen. |
| 4 | I would recommend digital medication reminder apps to my patients. | Ich würde meinen Patienten digitale Anwendungen zur Erinnerung an die Medikation empfehlen. |
| 5 | I would recommend information and education portals to my patients. | Ich würde meinen Patienten Informations- und Aufklärungsportale empfehlen. |

1. DIGAs are digital health applications that can be prescribed by physicians for patient use for a variety of diagnoses. [↑](#footnote-ref-1)
