# Supplementary material for: Understanding Dermatologists’ Acceptance of Digital Health Interventions: Cross-Sectional Survey and Cluster Analysis
Source: JMIR Hum Factors. 2025 May 21;12:e59757. doi: 10.2196/59757 (PMC12118942; doi:10.2196/59757)
Supplement: Multimedia Appendix 2 [file humanfactors-v12-e59757-s002.docx]

| Item number | Item | Factor loading | Missing  N (%) |
| --- | --- | --- | --- |
| Factor 1: Positive expectancies and acceptability of DHIs (alpha: 0.93); percent of variance explained: 45.9% | | | |
| 23 | DHIs can help transfer more responsibility to patients. | 0.729 | 4 (2.4) |
| 18 | DHIs can help me to complement the existing patient care in a meaningful way. | 0.687 | 4 (2.4) |
| 1 | I could imagine using more DHIs for the care of my patients. | 0.678 | 1 (0.6) |
| 16 | DHIs can help me make the consultation more demand-oriented. | 0.667 | 5 (2.9) |
| 19 | I would recommend DHIs to my patients. | 0.647 | 4 (2.4) |
| 12 | I would trust the recommendations of doctors in my immediate surroundings regarding DHIs. | 0.624 | 4 (2.4) |
| 11 | I would trust the recommendations of my professional association regarding DHIs. | 0.611 | 4 (2.4) |
| 17 | DHIs can help me improve doctor-patient communication. | 0.551 | 6 (3.5) |
| 2 | I would be willing to pay for or invest in DHIs. | 0.477 | 1 (0.6) |
| 27 | I could imagine discussing digitally collected data from the patient in the consultation session. | 0.475 | 0 (0.0) |
| Factor 2: Dermatologists’ digital competencies (alpha: 0.90); percent of variance explained: 9.5% | | | |
| 26 | I have good knowledge in digital medicine. | -0.797 | 0 (0.0) |
| 3 | I would find it easy to use a digital application. | -0.797 | 1 (0.6) |
| 24 | I have excellent skills in using digital media (e.g., PC. smartphone and tablet). | -0.777 | 4 (2.4) |
| 25 | I am personally interested in the topic of digital medicine. | -0.641 | 0 (0.0) |
| 5 | I could easily integrate DHIs into my daily work routine. | -0.504 | 1 (0.6) |
| Factor 3: Negative expectancies and barriers of DHIs (alpha: 0.73); percent of variance explained: 7.1% | | | |
| 15 | DHIs can increase the time required for patient care. | 0.779 | 6 (3.5) |
| 9 | The IT effort makes me hesitant to use DHIs. | 0.541 | 4 (2.4) |
| 14 | DHIs can quickly lead to information overload for me. | 0.508 | 4 (2.4) |
| 10 | The use of DHIs is currently not adequately reimbursed. | 0.495 | 4 (2.4) |
| Factor 4: Dermatologists' perspectives on patients’ acceptability and competencies (alpha: 0.80); percent of variance explained: 5.1% | | | |
| 20 | My patients would welcome the use of DHIs. | 0.943 | 6 (3.5) |
| 21 | It would be easy for my patients to use a DHI. | 0.669 | 4 (2.4) |
| Excluded variables due to low factor loadings < 0.4 | | | |
| 22 | DHIs can make patients feel insecure about their condition. | 0.396 | 7 (4.1) |
| 6 | My practice/clinic has the necessary infrastructure for the use of DHIs. | 0.389 | 0 (0.0) |
| 8 | I am concerned about misuse of collected data. | 0.386 | 4 (2.4) |
| 4 | I find it difficult to distinguish serious from dubious DHIs. | 0.308 | 0 (0.0) |
| 13 | My non-physician colleagues in my practice/clinic would welcome the use of DHIs. | 0.302 | 4 (2.4) |
| 7 | Data collected from the patient should be easily transferable to my records. | 0.297 | 1 (0.6) |
| Kaiser-Meyer-Olkin measure of sampling adequacy: 0.92  Bartlett’s Test of Sphericity: X² (df: 210) = 2264, *P* < 0.001  Cumulative percent of variance explained: 67.6% | | | |
